# A genome-wide survey of interaction between rice and *Magnaporthe oryzae* via microarray analysis

**DOI:** 10.1080/21655979.2020.1860479

**Published:** 2020-12-28

**Authors:** Yanping Tan, Xiaolin Yang, Minghao Pei, Xin Xu, Chuntai Wang, Xinqiong Liu

**Affiliations:** a Hubei Provincia Key Laboratory for Protection and Application of Special Plant Germplasm in Wuling Area of China, Key Laboratory of State Ethnic Affairs Commission for Biological Technology, College of Life Science, South-Central University for Nationalities, Wuhan, China; b Hubei Key Laboratory of Crop Disease, Insect Pests and Weeds, Institute of Plant Protection and Soil Science, Hubei Academy of Agricultural Sciences, Wuhan, China

**Keywords:** Rice, *Magnaporthe oryzae*, microarray, interaction

## Abstract

The main aim of the work is to study the regulation of gene expression in the interaction between rice and *Magnaporthe oryzae* by gene chip technology. In this study, we mainly focused on changes of gene expression at 24, 48, and 72 hours post-inoculation (hpi), through which we could conduct a more comprehensive analysis of rice blast-related genes in the process of infection. The results showed that the experimental groups contained 460, 1227, and 3937 significant differentially expressed genes at 24, 48, and 72 hpi, respectively. Furthermore, 115 significantly differentially expressed genes were identified in response to rice blast infection at all three time points. By annotating these 115 genes, they were divided into three categories: metabolic pathways, proteins or enzymes, and organelle components. As expected, many of these genes were known rice blast-related genes; however, we discovered new genes with high fold changes. Most of them encoded conserved hypothetical proteins, and some were hypothetically conserved genes. Our study may contribute to finding new resistance genes and understanding the mechanism of rice blast development.

## GRAPHICAL ABSTRACT

**Figure:**
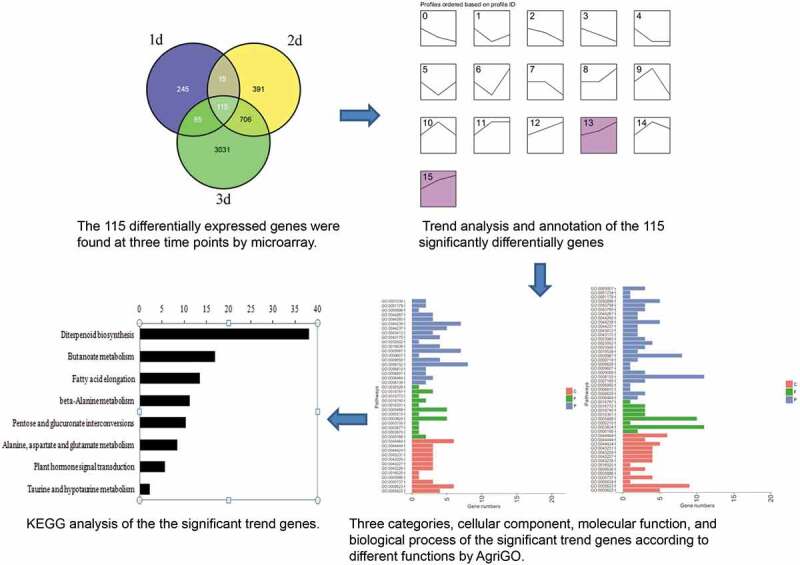

## Introduction

Rice is one of the most important food crops in the world, and it feeds more than 50% of the global population [1]. According to the United Nations Food and Agriculture Organization’s report in 2009, the world’s population will expand from 7.5 to 10.5 billion by 2050, especially in rice consuming countries [2]. The rice production will have to increase by 70% to match the current daily consumption level. Although rice production has increased during the last few decades, a large percentage of rice crops are being lost due to different abiotic and biotic stresses [1]. Rice blast, caused by the fungus *Magnaporthe oryzae*, is one of the most devastating diseases in rice and can lead to decreased rice production as much as 10%–50%. Therefore, researchers are trying to find the most effective method to solve this problem. The traditional method is to use chemical fungicides to prevent rice blast, but its high cost and serious pollution cannot meet the needs of modern social development. Currently, the most economical and effective way to control rice blast is to breed resistant varieties by biological methods. Cloning a new resistance gene with broad-spectrum resistance and understanding its function has been the main focus of current research and has met with some success, with many new resistance genes having been identified and cloned. In addition, many genes that are induced by rice blast, such as *PR1b, JAmyb*, and *PAL*, have been also found. By now, at least 84 resistance genes have been reported, such as R genes. These genes are located in the clusters of all chromosomes except Chr3. These resistance gene coding proteins have specific domains, and most of the resistance genes encode N end curly coiled-coil spiral domain, nucleotide binding sites and the C end with leucine-rich repeats (CC-NBS-LRR) [3,4].

Besides resistance genes, rice itself also has other ways to fight against rice blast. In order to defend against pathogens, plants have evolved a variety of immunity systems [5]. The first layer is called a congenital immune response. It refers to the identification of pathogens by recognition of receptors, called PAMP/MAMP-triggered immunity (PTI). PTI is resistant to the invasion of most potential pathogens. The second-generation congenital defense system is activated by the host’s R gene recognition Avr effector, called effector-triggered immunity (ETI). ETI is more rapid and potent than PTI and is usually associated with HR to inhibit the spread of pathogens [6]. However, these mechanisms can only prevent rice blast infection to a certain extent. Pathogens have evolved a variety of mechanisms to escape or interfere with PTI in a long-term struggle with plants. In the process of infection, the pathogens usually produce a specific effect or slip into the host cells to inhibit or interfere with the plant’s defense system.

In recent years, with the rapid development of bioinformatics and functional genomics, gene chip technology can handle large amounts of information, including large-scale DNA sequence analysis, comparison, and identification of gene functions [7]. Currently, gene chip technology is mainly used in the field of medicine [8,9]. Rice gene expression profile analysis has also been applied. The use of gene chip technology in rice disease research is advantageous to clarify the mechanism of disease resistance. At present, the gene chip method has been used to study the dynamic changes of the whole genome in the process of rice blast infection. Recently, a microarray study compared gene expression levels in *M. oryzae* mycelium grown under different stress conditions with those of the fungus invading the plant [10]. However, while these studies have analyzed the expression level of rice blast-related genes, they just focused on the gene expression levels at relatively late stages of infection, 3–10 days post-inoculation (dpi) [11], which was too late to investigate the expression level of genes in early stages. In addition, other studies also only focused on changes in the expression level of genes after 24 hpi, which is the time point of initial rice blast infection. While genes related to the defense systems are activated during this period, many rice blast-related genes are also activated in later stages.

In order to explore new resistance resources, and find the signal pathways involved in resistance response, our main goals were to focus on the dynamic changes of the genes during the 24–72 hpi to conduct a more comprehensive analysis of rice blast-related genes in the infection process. Through this method, some genes related to rice blast resistance and defense were further analyzed and verified. Some examples are the cell recognition-related resistance genes, NBS-LRR family genes and WAK kinase genes, the signal transduction-related genes, serine/threonine protein kinase family genes, as well as the transcriptional regulation-associated genes, the WRKY family, and MYB family genes [12–14]. Through our research, we expected to find some genes that are closely related to rice blast infection that were ignored in previous studies. Based on the results, this study might provide reference and data support for discovering new rice blast resistance genes.

## Materials and methods

### Rice material

The rice materials Nipponbare was used in this experiment. All the seeds were in good condition and then peeled. The seeds were sterilized with 2% sodium hypochlorite solution for three times in a constant temperature shaker. After sterilization, the seeds were washed by distilled and sterile water and then transferred to the previously prepared MS medium. Two weeks after germination, the seedlings were moved to soil until they grew to four-leaf stage.

### Magnaporthe oryzae

In this study, rice blast strain race 007 was used. The strain was cultivated at 25°C in oatmeal agar plates for 7–10 days. Afterward, plates were placed under purple light at 28°C for 3 days. During this period, many spores were produced on the surface of the oatmeal agar plates. The spores were washed with distilled water to form a suspension, and the spore concentration was adjusted to about 1.5 × 10^5^/mL. Infected samples were inoculated with the spore suspension by spraying, while the control samples were treated with distilled and sterile water. After inoculation, infected samples were placed in darkness for 24 h with 90% air humidity. The rice leaves were collected at 1 d, 2 d, and 3 d after inoculation. The weight of each rice leaf was about 100 mg of 3 repeats, and the leaves were stored in a − 80°C refrigerator immediately.

### Gene chip hybridization experiment

A total of 18 leaf samples were prepared for gene expression microarray experiments. Total RNA was extracted from leaves using TRIzol reagent (Invitrogen). Total RNA was subjected to quality control using a NanoDrop ND-2000 spectrophotometer (WI53711, Thermo Fisher, USA) and an Agilent Bioanalyzer 2100 (Agilent Technologies, Santa Clara, CA, US). Qualified RNAs were subjected to subsequent chip experiments. Agilent 4 × 44 K rice oligo microarray with 42,489 60-mer oligonucleotide probes was used for expression profile analysis. Twenty-four microarrays of single dye with three independent replicates for each time point were performed according to the manufacturer’s protocol at Shanghai Biotechnology Corporation.

Data filtering was conducted with the criteria that at least 3 out of all tested samples have P flag to ensure the quality of the normalized data. Student’s T test was performed between each of two groups. The differential expression genes were screened if the P-valueless than 0.05 in at least two groups on each of the time course. Hierarchical Cluster (HCL) incorporated in MeV (http://www.tm4.org/mev.html) was performed to get heatmap. STEM software [PMID: 16597342] was used to identify significant temporal expression profiles and the genes associated with these profiles and to compare the behavior of these genes across time course.

The gene classification based on gene ontology was conducted at AgriGO database (http://bioinfo.cau.edu.cn/agriGO/). The pathway analysis was performed with MapMan [PMID: 14996223] after converting Probe_ID into MSU_ID according to the annotation from Rice Genome Annotation Project (http://rice.plantbiology.msu.edu/).

### Quantitative real-time PCR analysis

We used 2 µg of total RNA to synthesize first-strand cDNA using random hexamers and a cDNA reverse transcription kit (TaKaRa, Japan) in a reaction volume of 20 μL. Real-time PCR was performed using an SYBR Premix Ex TaqTM Kit (TaKaRa) on an ABIprism 7500 Real-Time PCR System (A28132, ABI, USA). A rice ubiquitin gene (Os03g0234200) was used as a reference gene with actin RNA serving as the internal control. The expression levels of samples were measured using the 2^−ΔΔCt^ method. All measurements were performed in triplicate, and the experiments were repeated at least twice.

## Results and discussion

### Screening of differentially expressed genes involved in the interaction between rice and *M. oryzae*

A large number of differentially expressed genes were obtained by comparing the experimental groups with the control groups at the corresponding time points. Genes with the expression level differing by more than 2-fold or less than 0.5-fold were regarded as differentially expressed genes. The results showed that the number of DEGs increased at three time points, with an obvious ascending trend. This trend suggests that a large number of genes are involved in the interaction between rice and rice blast, making it a complex and redundant process. Compared with the control groups, the experimental groups showed 460, 1227, and 3937 significant DEGs at 24, 48, and 72 h after inoculation, respectively (Figure. S1). These data further demonstrated that analyzing DEGs at 24 or after 72 hpi and not at 48 hpi was not comprehensive. It was worth noting that the expression of 115 genes had significant differences at all the three time points, suggesting that these genes might be highly related to the response of rice to rice blast infection (Figure. S1).

**Figure 1.**
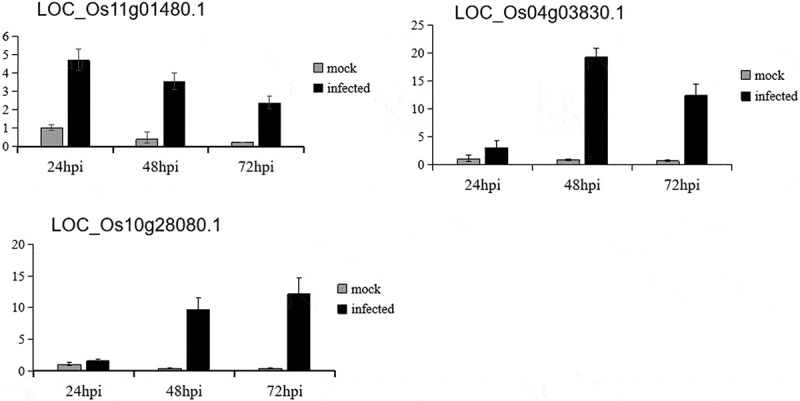
The expression level of the genes chosen for confirmation by QRT-PCR at three time points

To verify the reliability of chip data, we randomly selected three differentially expressed genes, representing the different components of the defense response, for real-time quantitative PCR analysis (Table 1). The signal transduction-related gene, Serine/threonine protein kinase-related domain-containing protein (LOC_Os04g03830.1),the transcriptional regulation-associated gene, MYB family transcription factor (LOC_Os11g01480.1), and the defense gene, chitinase 1 precursor (LOC_Os10g28080.1), all showed significantly different expression at three time points, ranging from 2-fold to 20-fold. Results from qRT-PCR showed similar results (Figure 1). These data suggested that the microarray data were reliable.

**Table 1.** Genes chosen for confirmation by qRT-PCR

Locus ID	Description	Fold change		
		24hpi	48hpi	72hpi
LOC_Os11g01480.1	MYB family transcription factor	2.3	3.8	10.5
LOC_Os10g28080.1	Similar to Chitinase 1 precursor	2.2	11.7	21.6
LOC_Os04g03830.1	Serine/threonine protein kinase-related domain containing protein.	2.2	3.0	11.0

### Trend analysis and annotation of the 115 significantly differentially expressed genes

**Figure 2.**
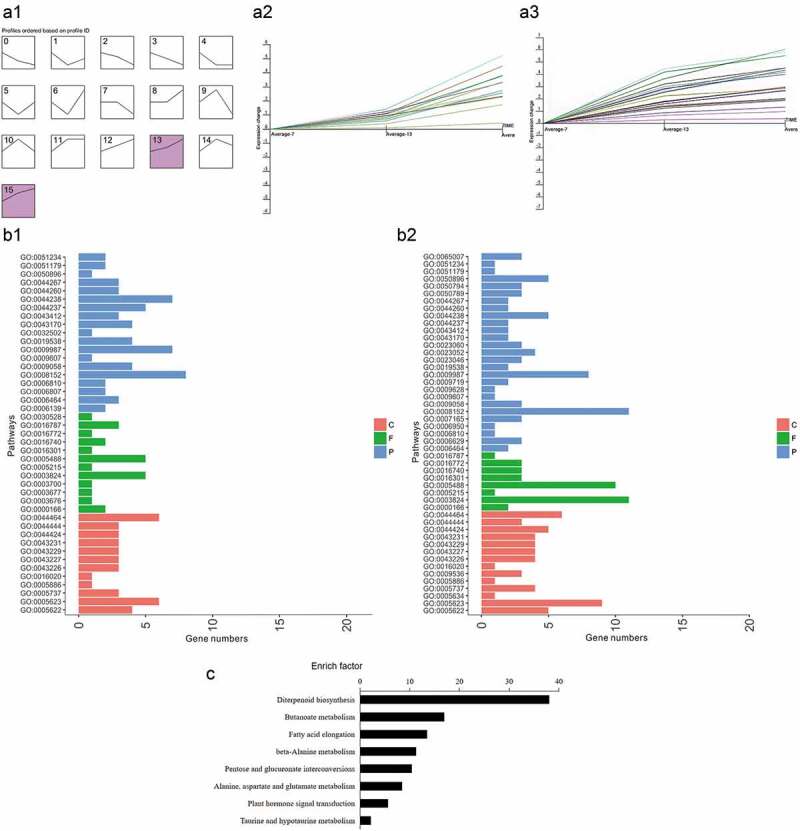
Trend analysis and annotation of the 115 significantly differentially expressed genes. (a) Using stem software to cluster the change trend of the 115 genes during the three time points. Marked color chart is a significant trend, such as the thirteenth and fifteenth diagram. (b) Three categories of the significant trend genes according to different functions by AgriGO database. C, F and P corresponding to cellular component, molecular function, and biological process, respectively. (c) KEGG analysis of the significant trend genes

Since the expression of these 115 genes was significantly different at all time points, analyzing these genes might help elucidate the response mechanism of rice to rice blast. We used bioinformatics to annotate and classify these genes and determine significance. Using stem software to analyze the trend of these genes, we found several significant changes in the class (P < 0.05). The color of the chart is a significance trend, which represents the trend of gene expression in the experiment. All genes can be divided into 16 trends. These 16 kinds of trends mainly include a continuous decline, a decline after a rise, rising, and rising and then falling. From the figure, we see that classes 13 and 15 were the main trends of these genes (Figure 2a), showing a continuous upward trend but with different slopes. After annotating these genes, we found that they could be divided into three major functional categories: metabolic pathways, proteins or enzymes, and organelle components (Figure 2b). KEGG analysis of these 115 genes showed that these genes are involved in various metabolic pathways including diterpenoid biosynthesis, butanoate metabolism, fatty acid elongation, beta-Alanine metabolism, pentose and glucuronate interconversions, alanine, aspartate and glutamate metabolism, plant hormone signal transduction, and taurine and hypotaurine metabolism (Figure 2c).

Many of the 115 genes are involved in diterpenoid biosynthesis. Previous studies have shown that rice can produce three types of secondary metabolites: terpenes, alkaloids, and phenylpropanoids. Diterpenoid phytoalexins are low-molecular-weight antimicrobial secondary metabolites that are primarily produced by plants to combat microbial pathogen infections [15] and are critical to resist fungal and bacterial pathogens [16]. The diterpenoid phytoalexins biosynthetic genes were barely upregulated in BTH-treated rice plants but were induced rapidly after *M. oryzae* infection [17,18]. Diterpenoid phytoalexins are considered important in the plant defense system against a variety of pathogens and as molecular markers of disease resistance [19–21].

### The expression of rice defense-related genes

In the 115 DEGs, we obtained several representative genes, which were associated with pathogen recognition, signal transduction, transcriptional regulation, and defense-related and biochemical metabolism (Table 2). Four of the genes involved in pathogen identification are OsWAK receptor-like protein kinase, which transmit signals directly by their cytoplasmic kinase domains. They are primarily involved in regulating plant cell wall functions [22], including cell expansion [23], binding and regulation of pectins [24], pathogen response, and protection of plants from detrimental effects [25]. There are also three NBS-LRR proteins involved in pathogen identification. The NBS-LRR domain is a typical domain of resistance genes and plays an important role in the defense against microbial invasion [3]. In addition, serine/threonine protein kinase plays an important role in disease resistance in rice. For example, *OsPBL1* plays an important role in defense response mainly by promoting the salicylic acid-dependent pathway to resist pathogen infection [26]. Cytochrome P450 family proteins are also involved in rice’s pathogenic resistance [27]. For example, *CYP71Z2* is a P450 gene that enhances the resistance of rice to pathogenic bacteria by regulating phytoalexin synthesis [20].

**Table 2.** The expression of rice defense-related genes

Locus ID	Description	Fold change		
		24hpi	48hpi	72hpi
Pathogen recognition				
LOC_Os04g30260.1	OsWAK47 – OsWAK receptor-like protein kinase	3.2	2.7	18.5
LOC_Os09g29510.1	OsWAK80 – OsWAK receptor-like protein kinase	2.0	3.4	11.4
LOC_Os04g29580.1	OsWAK37 – OsWAK short gene	2.7	2.2	16.1
LOC_Os04g30250.3	wall-associated receptor kinase-like 5 precursor	2.9	2.1	6.3
LOC_Os11g11960.1	Similar to NBS-LRR disease resistance protein homologue	2.2	2.6	10.6
LOC_Os11g12320.1	Similar to NBS-LRR protein	2.0	2.2	11.3
LOC_Os11g12040.1	Similar to NBS-LRR protein	2.0	2.1	5.6
LOC_Os06g10790.1	lectin-like receptor kinase	3.2	2.4	8.0
Signal Transduction				
LOC_Os01g50420.1	Serine/threonine protein kinase domain containing protein	0.2	76.4	829.3
LOC_Os04g03830.1	Serine/threonine protein kinase-related domain containing protein	2.2	3.0	11.1
Transcriptional regulation				
LOC_Os11g01480.1	Myb-like DNA-binding domain, SHAQKYF class family protein	2.3	3.8	10.5
LOC_Os08g36920.1	Similar to AP2 domain containing protein RAP2.6	0.1	16.6	136.7
Defense				
LOC_Os10g28080.1	Similar to Chitinase 1 precursor	2.2	11.7	21.6
Biochemical metabolism				
LOC_Os12g24320.1	ATPase 3, AAA-type, core domain containing protein	3.2	15.6	246.6
LOC_Os01g38110.1	Cytochrome P450 family protein	2.3	21.7	53.5
LOC_Os12g39310.1	Cytochrome P450 family protein	0.2	3.5	31.8
LOC_Os08g39730.1	Oryza sativa Cyt-P450 monooxygenase (PM-II) mRNA, complete cds	0.4	16.7	8.7
LOC_Os04g10160.1	Similar to Cytochrome P450 CYP99A1	5.0	28.9	154.6

The expression levels of these genes at 24 hpi were consistent with those obtained in previous studies, and most of them were up-regulated, with a few showing a downward trend. With the addition of the third time point, we were able to show an up-regulation trend of these genes. For example, most of the pathogen recognition genes showed a 2-fold increase in 24 hpi and 48 hpi, but their expression increased to 10-fold or higher at 72 hpi. In addition, the expression levels of genes associated with biochemical and metabolic processes were significantly up-regulated to about 20-fold at 48 hpi, and at 72 hpi rose to more than 100-fold, which was the case for the expression level of ATPase 3 (246.6-fold up-regulation at 72 hpi). It was worth noting that some of the genes down-regulated at 24 hpi showed a significant up-regulation trend at the later time points. Examples include serine/threonine protein kinase domain-containing protein (LOC_Os01g50420.1), similar to AP2 domain-containing protein RAP2.6 (LOC_Os08g36920.1), cytochrome P450 family protein (LOC_Os12g39310.1), and Oryzae sativa Cyt-P450 monooxygenase (LOC_Os08g39730.1). The expression levels of the genes above were reduced to about 0.2-fold at 24 hpi but increased to 10-fold or more from 48 hpi to 72 hpi, which was not consistent with the result of the previous study. Because of the significant changes these genes display, they may play an important role in the interaction between rice and rice blast. Overall, our experimental results reveal a more comprehensive analysis of the rice blast-related genes in the process of infection. Previous studies mainly focused on the microarray analysis of the genes for 24 hpi. Rice blast infected rice mainly at this time point, and most of the resistance-related genes responded to infection at this time. However, the response of some genes may be delayed, so we studied 115 genes with significant changes in 24, 48, and 72 hpi. Through this method we could also reduce the interference of genes that only function at 24 hpi. As expected, we obtained many genes related to rice blast resistance among the 115 genes, such as OsWAK receptor-like protein kinase, NBS-LRR protein, serine/threonine protein kinase domain-containing proteins, and cytochrome P450. They are involved in transcriptional regulation and biochemical metabolism. In addition, we also obtained hypothetical proteins and genes with significant fold changes. Most of them showed a significant increase in expression at three time points, indicating that they are involved in the interaction between rice and *M. oryzae*. Our results provide data to help understand the interaction mechanism of rice blast with rice and also provide a reference for discovering new resistant genes.

### The discovery of new rice blast-related genes

In addition to the annotated genes mentioned above, we also obtained several genes that have not yet clearly defined. Most of them encoded conserved hypothetical proteins, while others were hypothetically conserved genes. Two of them were function-unknown proteins (Table 3). Conserved hypothetical proteins are obtained by sequencing, which generally contain certain domains that are conserved in many species and may have some important functions that are not yet determined [28,29]. The expression of genes or proteins in response to rice blast showed a significant change, suggesting their involvement in rice blast infection at different stages with different expression levels [30–32]. Most of the conserved hypothetical proteins or hypothetical conserved genes, as well as the two DUF proteins, showed a sustained increase in expression. For example, conserved hypothetical protein LOC_Os06g38660.1 continued to increase from 2- to 200-fold during the three time points. Some genes showed a tendency of being up-regulated first and then down-regulated. For example, the expression level of LOC_Os09g17146.1 and LOC_Os12g05690.1 was down-regulated to 0.5-fold at 24 hpi but later increased to over 100 fold. Only a few genes showed continuous down-regulation. Because of the significant fold change in many of the hypothetical proteins and genes, further studies should be pursued to discover possible new resistance genes among them.

**Table 3.** The expression of rice novel defense-related genes

Locus ID	Description			Fold change		
				24hpi	48hpi	72hpi
LOC_Os01g02130.1		Conserved hypothetical protein		2.2	2.8	13.5
LOC_Os01g48620.1			Conserved hypothetical protein	2.7	2.3	48.1
LOC_Os01g60930.1			Conserved hypothetical protein	2.89	2.69	8.89
LOC_Os06g38660.1			Conserved hypothetical protein	2.2	18.6	221.0
LOC_Os09g04310.1			Conserved hypothetical protein	2.7	3.3	15.0
LOC_Os12g31540.1			Conserved hypothetical protein	3.5	2.8	18.5
LOC_Os04g03164.1			Hypothetical conserved gene	2.2	6.5	36.9
LOC_Os04g22120.1			Hypothetical conserved gene	2.9	6.4	13.2
LOC_Os07g31190.1			Hypothetical conserved gene	2.7	2.7	12.0
LOC_Os11g38790.2			Hypothetical conserved gene	3.0	4.7	58.4
LOC_Os07g03040.2			Protein of unknown function DUF1719, Oryza sativa family protein	2.77	7.07	53.0
LOC_Os12g33300.1			Protein of unknown function DUF6, transmembrane domain containing protein	2.2	2.7	26.4
LOC_Os01g09220.1			Conserved hypothetical protein	0.2	2.6	15.1
LOC_Os01g50350.2			Conserved hypothetical protein	0.2	6.1	27.8
LOC_Os01g62190.1			Conserved hypothetical protein	0.3	6.1	23.8
LOC_Os05g47960.1			Conserved hypothetical protein	0.3	3.5	9.2
LOC_Os09g17146.1			Conserved hypothetical protein	0.5	3.5	149.2
LOC_Os12g05690.1			Conserved hypothetical protein	0.4	9.3	108.2
LOC_Os06g02830.1			Hypothetical conserved gene	0.3	0.4	0.2
LOC_Os03g08980.1			Conserved hypothetical protein	3.5	2.9	0.4
LOC_Os03g10250.1			Conserved hypothetical protein	0.4	6.5	0.1

## Conclusions

Fungus *M. oryzae* is known to cause rice blast, which is one of the most harmful diseases in rice. Because of the variability of *M. oryzae*, the resistance gene is easy to lose resistance. Therefore, it is the best way to explore more broad-spectrum resistance resources for disease resistance breeding and analyze the disease resistance mechanism. In order to further understand the interaction between rice and *M. oryzae*, we analyzed the changes of gene expression in rice post inoculation. Based upon the comprehensive analysis, we identified 460, 1227, and 3937 genes that are significantly changed in expression within experimental groups, at 24, 48, and 72 hpi, respectively. Interestingly, all three time points shared 115 genes that are significantly changed in expression upon *M. oryzae* inoculation. Functional Gene Groups analysis have divided these 115 genes into three categories, most of which were known to be rice blast related. However, we discovered some new genes that were changed with high level of statistical significance. Some of these new genes encoded hypothetically conserved genes, while the rest mostly encoded conserved hypothetical proteins. In conclusion, our research contributes to further understanding of the molecular mechanisms underlying rice blast development and may lead to the finding of new resistance genes.

## Supplementary Material

Supplemental Material

## Highlights

- Some new signaling pathways involved in blast disease resistance are revealed.
- Many new rice defense-related genes are found to involve in interaction between rice and *M. oryzae*.
- Many conserved hypothetical proteins with unknown functions are found to involve in blast disease resistance.
