# Supplementary material for: A genome-wide survey of interaction between rice and Magnaporthe oryzae via microarray analysis
Source: Bioengineered. 2020 Dec 28;12(1):108–16. doi: 10.1080/21655979.2020.1860479 (PMC8806351; doi:10.1080/21655979.2020.1860479)
Supplement: Supplemental Material [file KBIE_A_1860479_SM2976.zip › supplementary/graphical abstract.pptx]

## Slide 1
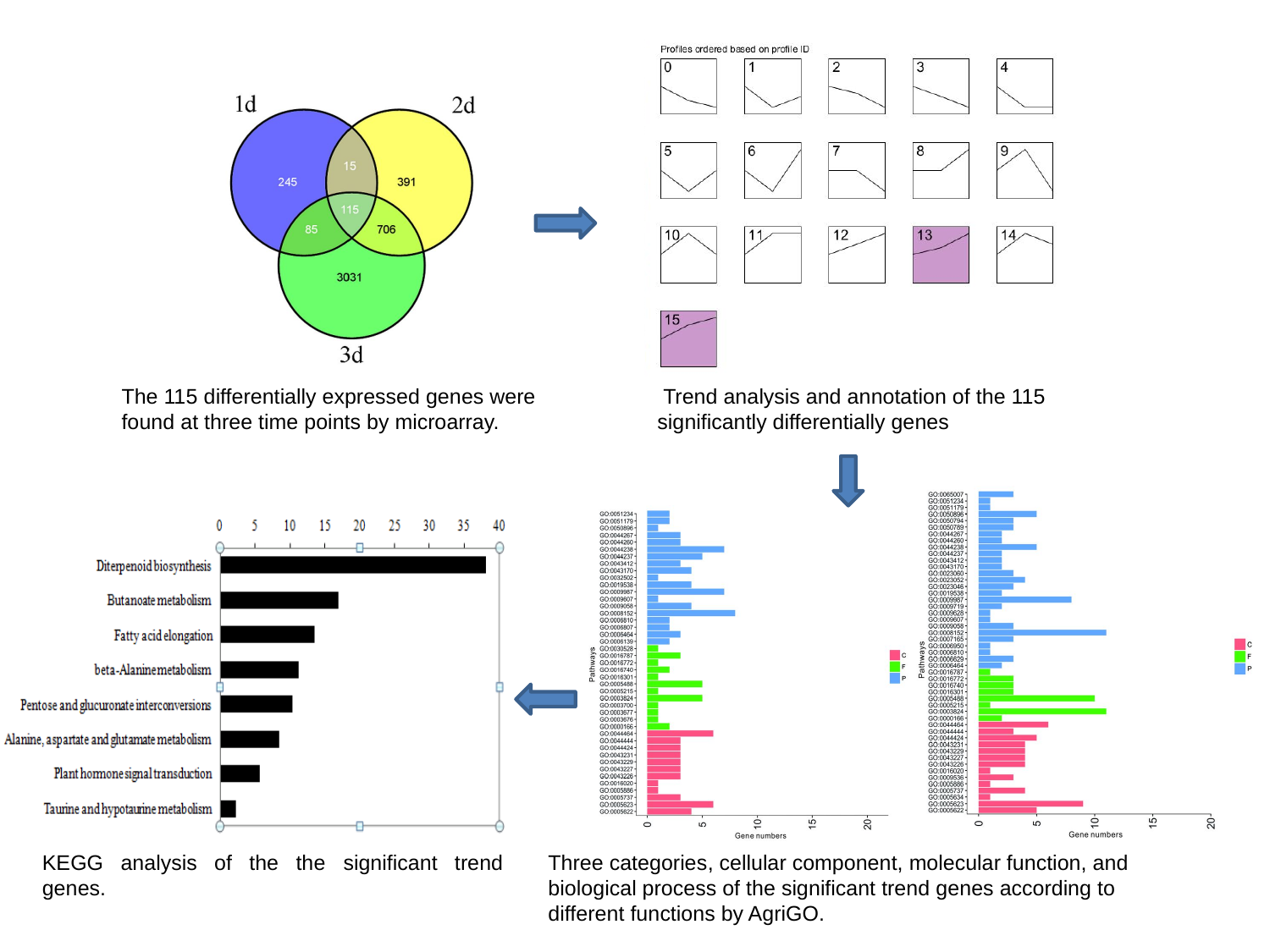

The 115 differentially expressed genes were found at three time points by microarray.
 Trend analysis and annotation of the 115 significantly differentially genes
KEGG analysis of the the significant trend genes.
Three categories, cellular component, molecular function, and biological process of the significant trend genes according to different functions by AgriGO.
