# Supplementary material for: A genome-wide survey of interaction between rice and Magnaporthe oryzae via microarray analysis
Source: Bioengineered. 2020 Dec 28;12(1):108–16. doi: 10.1080/21655979.2020.1860479 (PMC8806351; doi:10.1080/21655979.2020.1860479)
Supplement: Supplemental Material [file KBIE_A_1860479_SM2976.zip › supplementary/Highlights.docx]

**Highlights**

- Some new signaling pathways involved in blast disease resistance are revealed.
- Many new rice defense-related genes are found to involve in interaction between rice and *M. oryzae*.
- Many conserved hypothetical proteins with unknown functions are found to involve in blast disease resistance.
