# Supplementary material for: A genome-wide survey of interaction between rice and Magnaporthe oryzae via microarray analysis
Source: Bioengineered. 2020 Dec 28;12(1):108–16. doi: 10.1080/21655979.2020.1860479 (PMC8806351; doi:10.1080/21655979.2020.1860479)
Supplement: Supplemental Material [file KBIE_A_1860479_SM2976.zip › supplementary/Upload supplementary files.docx]

**Figure S1. Differentially Expressed Genes at three time points.** The experimental group and the control group were collected at 24, 48 and 72 hours after inoculation, and the expression of genes in both groups were analyzed by microarray in order to get the significantly differentially expression genes (DEGs).
