# Supplementary figures and images for: A genome-wide survey of interaction between rice and Magnaporthe oryzae via microarray analysis
Source: Bioengineered. 2020 Dec 28;12(1):108–16. doi: 10.1080/21655979.2020.1860479 (PMC8806351; doi:10.1080/21655979.2020.1860479)

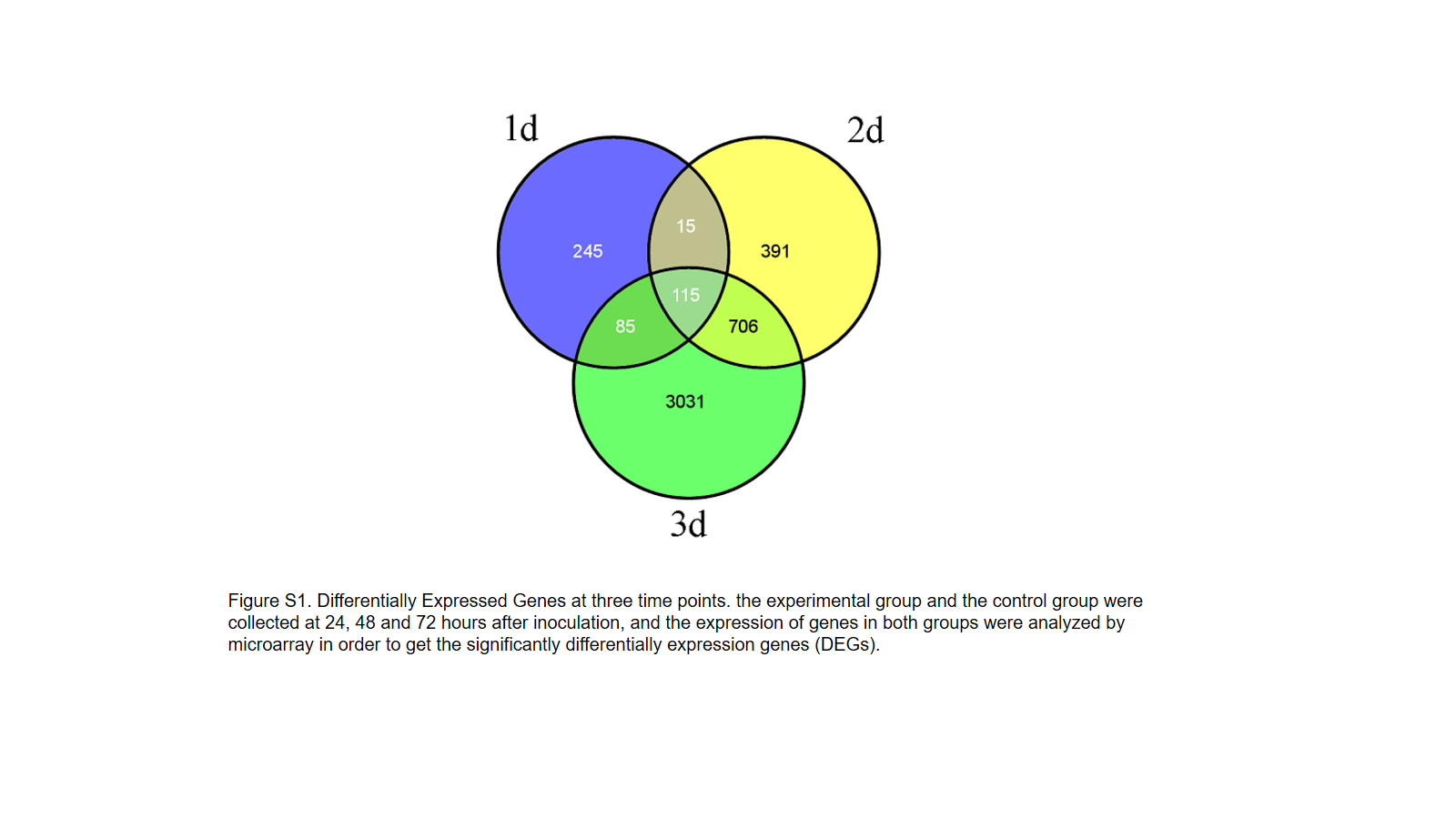

Supplement: Supplemental Material [file KBIE_A_1860479_SM2976.zip › supplementary/Fig S1.tif]
